# Fast 3D Super-Resolution Ultrasound with Adaptive Weight-Based Beamforming

**DOI:** 10.1109/TBME.2023.3263369

**Published:** 2023-03-30

**Authors:** Jipeng Yan, Bingxue Wang, Kai Riemer, Joseph Hansen-Shearer, Marcelo Lerendegui, Matthieu Toulemonde, Christopher J Rowlands, Peter D. Weinberg, Meng-Xing Tang

**Affiliations:** Ultrasound Lab for Imaging and Sensing, Department of Bioengineering, Imperial College London, London, UK, SW7 2AZ; Ultrasound Lab for Imaging and Sensing, Department of Bioengineering, Imperial College London, London, UK, SW7 2AZ; Ultrasound Lab for Imaging and Sensing, Department of Bioengineering, Imperial College London, London, UK, SW7 2AZ; Ultrasound Lab for Imaging and Sensing, Department of Bioengineering, Imperial College London, London, UK, SW7 2AZ; Ultrasound Lab for Imaging and Sensing, Department of Bioengineering, Imperial College London, London, UK, SW7 2AZ; Ultrasound Lab for Imaging and Sensing, Department of Bioengineering, Imperial College London, London, UK, SW7 2AZ; Department of Bioengineering, Imperial College London, London, UK, SW7 2AZ; Department of Bioengineering, Imperial College London, London, UK, SW7 2AZ; Ultrasound Lab for Imaging and Sensing, Department of Bioengineering, Imperial College London, London, UK, SW7 2AZ

**Keywords:** Ultrasound localisation microscopy (ULM), super-resolution, contrast-enhanced ultrasound, 3D beamforming

## Abstract

**Objective:**

Super-resolution ultrasound (SRUS) imaging through localising and tracking sparse microbubbles has been shown to reveal microvascular structure and flow beyond the wave diffraction limit. Most SRUS studies use standard delay and sum (DAS) beamforming, where high side lobes and broad main lobes make isolation and localisation of densely distributed bubbles challenging, particularly in 3D due to the typically small aperture of matrix array probes.

**Method:**

This study aimed to improve 3D SRUS by implementing a new fast 3D coherence beamformer based on channel signal variance. Two additional fast coherence beamformers, that have been implemented in 2D were implemented in 3D for the first time as comparison: a nonlinear beamformer with *p*-th root compression and a coherence factor beamformer. The 3D coherence beamformers, together with DAS, were compared in computer simulation, on a microflow phantom and *in vivo*.

**Results:**

Simulation results demonstrated that all three adaptive weight-based beamformers can narrow the main lobe, suppress the side lobes, while maintaining the weaker scatter signals. Improved 3D SRUS images of microflow phantom and a rabbit kidney within a 3-second acquisition were obtained using the adaptive weight-based beamformers, when compared with DAS.

**Conclusion:**

The adaptive weight-based 3D beamformers can improve the SRUS and the proposed variance-based beamformer performs best in simulations and experiments.

**Significance:**

Fast 3D SRUS would significantly enhance the potential utility of this emerging imaging modality in a broad range of biomedical applications.

## I Introduction

Super-resolution (SR) ultrasound (US) also known as ultrasound localisation microscopy (ULM) can, through localisation and tracking of microbubbles [1], [2], reconstruct images of the microvasculature with resolution beyond the diffraction limit in 2D [3]–[11] and 3D [12]–[15]. Compared to 2D, 3D imaging can offer volumetric coverage with super-resolution in all three dimensions, and does not have the out-of-plane motion that 2D imaging suffers from. However, the image quality of 3D US is generally not as good as 2D. Matrix array probes used for generating 3D images require significantly more elements than comparable 1D array probes. Matrix array probes often are manufactured with a smaller footprint to reduce channel count whilst maintaining sufficient pitch. Consequently, the main lobe of the point spread function (PSF) in 3D US is usually wider than in 2D US. Smaller apertures also limit the improvement of coherent compounding as effective steering angle range is reduced as overlapping region of isonification is reduced. Row-Column Array (RCA) probes have been designed for 3D US with larger apertures and fewer channels than matrix array probes [16]–[19], but the long element causes strong side lobes and distort the PSF in the nearfield/close to the probes.

The acquisition time of SRUS based on localising sparse microbubbles heavily depends on the number of separable bubbles within each image frame and desired completeness of reconstruction [20]–[22]. Imaging with wide main lobes makes it difficult to isolate close bubbles, while strong side lobes can increase the possibility of mistaking the side lobes as distinct bubbles. Using a lower concentration of bubbles and higher detection thresholds can mitigate these complications. This approach, however, significantly reduce the number of bubbles that can be localised from one frame, which will in turn increase the acquisition duration. Exploring methods to reduce the size of the PSF main lobe and the amplitude of the side lobes is thus valuable.

The sizes of the PSF main lobe and side lobes are affected by the transducer aperture size and by the beamforming algorithm used. Delay and Sum (DAS) is the most commonly used beamformer for high-frame-rate US, especially for 3D US [14], [15], [23], [24]. Apodisation can reduce PSF side lobes at the expense of an enlarged main lobe. In 2D US, coherence-based beamformers have been demonstrated to be able to reduce PSF main lobe widths and side lobe heights and improve image quality generally when compared to DAS beamforming [25]. There are several types of coherence-based beamformers. Some use different ways of estimating the coherence among channels. For example, the delay multiply and sum (DMAS) beamformer [26] calculates the sum of multiplications between coupled signals and short-lag spatial coherence beamformers [27] calculates the spatial covariance across the receive aperture. Some adaptively changes the weights for each channel signals before summing them, such as the minimum variance (MV) [28], [29] and the nonlinear beamforming with *p*-th root compression (*p*-DAS) beamformers [30]. Some, such as the coherence factor (CF) beamformer [31], applys adaptive weights to pixels. These advanced beamformers present significant opportunities to improve SRUS imaging in 3D, but also pose challenges due to the number of channels acquired and quantity of pixels that need to be reconstructed for one 3D US frame.

Adaptive weight-based beamformers have only incremental computational costs compared to the standard DAS beamformer. They are promising for 3D SRUS as their superiority over the DAS beamformer has already been demonstrated on 2D US. The CF beamformer is widely used and the *p*-DAS beamformer has been proven to outperform other kinds of adaptive weight-based beamformer [25], such as phase coherence and sign coherence [32]. Therefore, *p*-DAS, CF and a computationally efficient 3D beamformer based on variance in the channel data are investigated in this study. They are evaluated through simulations and experiments.

## II Methods

In this section the different beamformers and their implementation are introduced, and this is followed by the description of the simulation and *in vitro* and *in vivo* experiments. All the coherence beamformers investigated in this study are based on the spatial coherence of channel signals from individual transducer elements. The beamfomers are implemented in CUDA (v10.0, Nvidia, CA, USA) and other data processing is done in MATLAB (2021a, MathWorks, MA, USA) on a desktop (CPU: AMD Ryzen 9 5900 Processor, GPU: Nvidia Geforce RTX3080, RAM: 128 Gb).

### A Delay and Sum

Delay and Sum beamforming (DAS) [33] reconstructs a pixel (*y*) by

(1) $$y(t)=\sum_{n=1}^{N}s_{n}(t)$$

where *s_n_*(*t*) is delayed signal for the *n*-th channel and *N* is the number of channels. To demonstrate the issues with 3D DAS, a matrix array probe (Vermon, Tours, France) with 32×32 elements, 7.8 MHz centre frequency and a 9.6 mm×10.2 mm aperture imaging one point scatter is simulated in Field II [34]. For envelope detection, a Hilbert transform is applied to each channel prior to reconstruction.

**Fig. 1:**
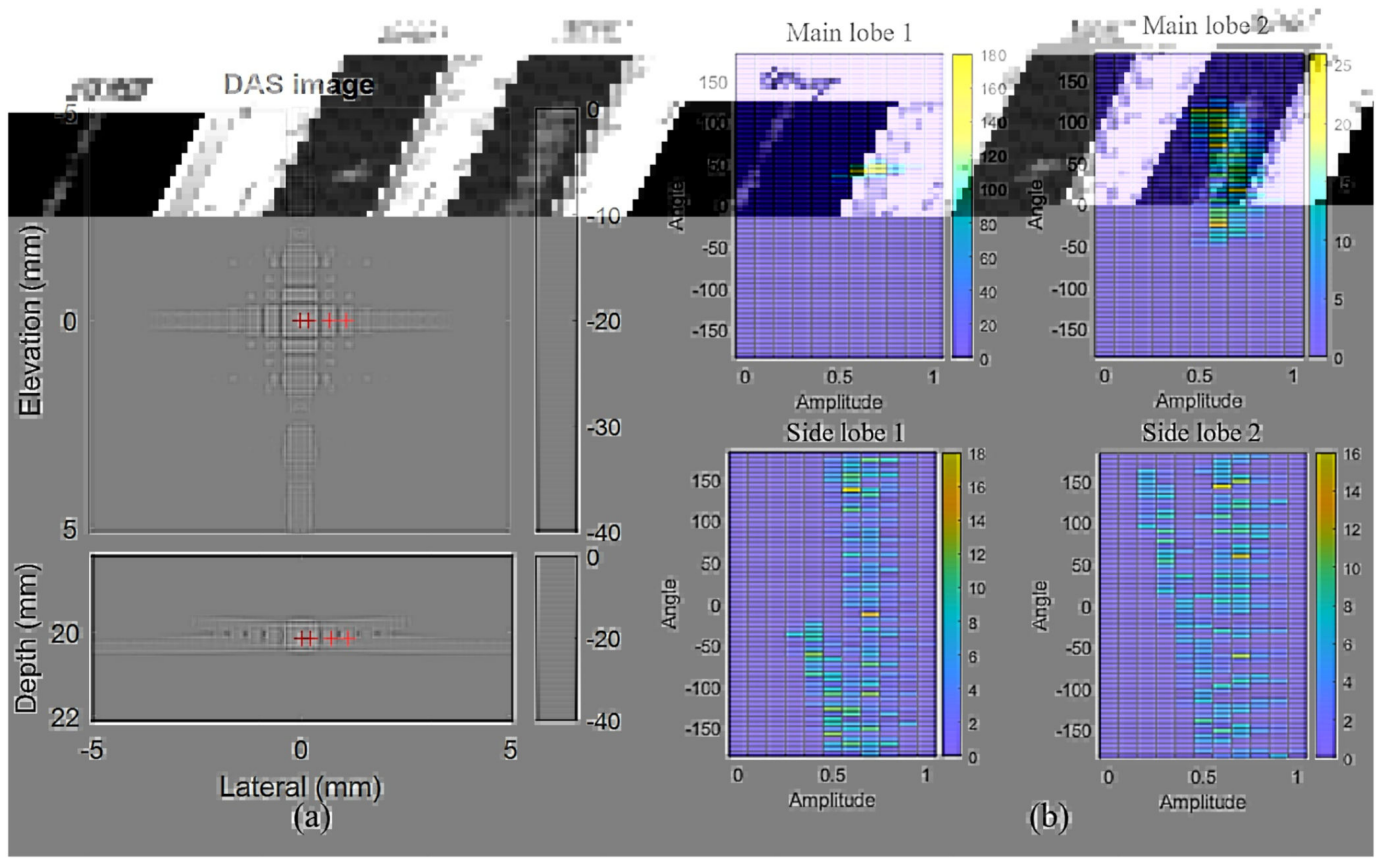
(a) B-mode image reconstructed by DAS. (b) Amplitude-angle histogram of delayed channel signals, where each pixel in the histogram is the number of channels with the same signal amplitude and phase angle. Four plots in (b) correspond to the four pixels labelled by red cross in (a). Note the colour bars have different ranges.

The brightness-mode (B-mode) 3D image of a point scatter reconstructed by DAS is shown in Fig. 1 (a). Four pixels are picked from the main lobe and side lobes, labelled by the red crosses, and denoted as main lobe 1-2, and side lobe 1-2 in order. Two-dimensional histograms of delay-cancelled signals corresponding to the four voxels are presented in Fig. 1 (b). Channel signals on the main lobe have similar amplitudes and phases, while channel signals on the side lobes have more dispersed amplitudes and phase angles. Therefore, summing up the channel signals would generate a main lobe brighter than the side lobes.

Element sensitivity apodisation *a_n_*(*t*), derived from the element directivity [35], is used for the DAS beamformer in the following comparison to reduce the intensity of side lobes. The DAS algorithm then becomes:

(2) $$\begin{array}{c} y(t)=\sum_{n=1}^{N}a_{n}(t)s_{n}(t)a_{n}(n)=\sin c\\ \sin c(\frac{Lu}{\lambda })\times \sin c(\frac{Lv}{\lambda })\times \cos(\theta )\\ u=\sin(\theta )\cos(\phi ),v=\sin(\theta )\sin(\phi ) \end{array}$$

where *L* is the width of the square element; λ is the wavelength; θ is the angle between the axial direction and the line connecting the element centre and pixel; φ is the angle between lateral direction and the projection of the line on the probe surface. Any channel with element sensitivity less than 0.5 for one voxel is not used for reconstructing the voxel in this paper.

### B Nonlinear Beamforming with p-th Root Compression

A nonlinear beamformer based on *p*-th root compression (*p*-DAS) [30] was designed by applying adaptive weights

(3) $$w_{n}(t)=\frac{1}{{|s_{n}(t)|}^{\frac{p-1}{p}}}$$

to channel signals followed by summation:

(4) $$\begin{array}{c} {\hat{y}}_{pDAS}(t)=\sum_{n=1}^{N}w_{n}(t)s_{n}(t)\\ =\sum_{n=1}^{N}\mathrm{sign}\\ \left(s_{n}\text{(t)}\right)\\ |s_{n}(t){|}^{1/p} \end{array}$$

where the sign function is used to maintain the phase of signals. Then, *ŷ_p_DAS*(*t*) is raised to the *p*-th power to recover the amplitude of the signal

(5) $$y_{pDAS}(t)=\mathrm{sign}({\hat{y}}_{pDAS}(t))|{\hat{y}}_{pDAS}(t){|}^{p}$$

As the *p*-th root and *p*-th power can distort the frequency components, spatial band-pass filtering around the fundamental spatial frequency is applied to the depth of *y_p_DAS*(*t*) to remove the distorted components. The sampling frequency along depth needs to be sufficient to avoid aliasing of artificial harmonics. In this study, *p* is set to 4, with which p-DAS generated the best lateral resolution among all the adaptive weight-based beamformers in [25].

### C Coherence Factor

The Coherence factor (CF) is defined by the ratio of the coherent energy to the incoherent energy

(6) $$W_{CF}(t)=\frac{|\sum_{n=1}^{N}s_{n}(t){|}^{2}}{\sum_{n=1}^{N}|s_{n}(t){|}^{2}}$$

*W_CF_* is calculated for each pixel and then used as an adaptive weight to the DAS image, i.e.,

(7) $$y_{CF}(t)=W_{CF}(t)y(t)$$

*W_CF_* approaches one when channel signals are phase coherent and it approaches zero if channel signals are completely incoherent. For example, the histograms of the main lobe 1 and side lobe 1 in Fig. 1(b) have a similar range of signal amplitude but a very different range of phase distribution. Applying CF weighting to the beamformer would therefore enhance the main lobe and reduce the side lobe.

### D Coherence Energy to Variance

**Fig. 2:**
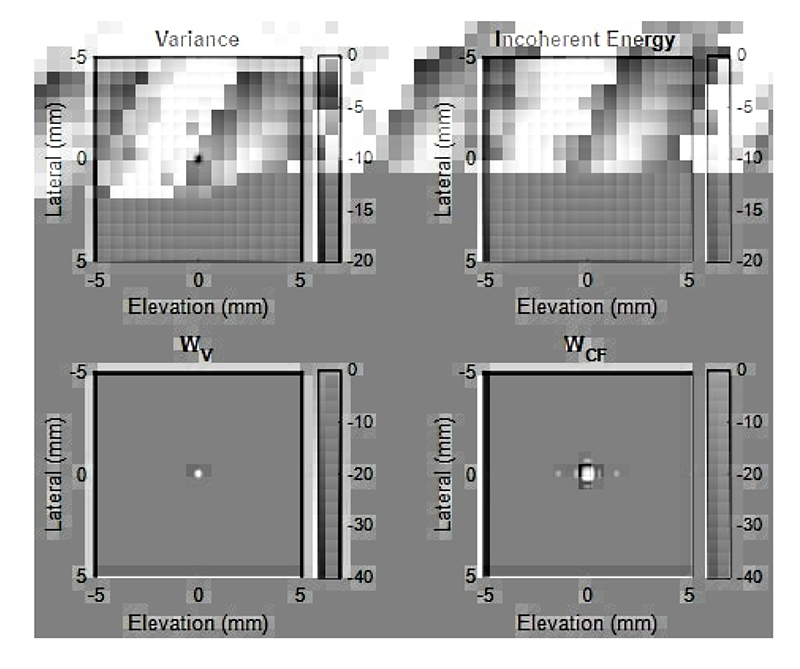
Demonstration of the principal difference between CF and CV beamformers. Images are plotted at the same lateral-elevation slice with the Fig. 1 (a). Each image is self-normalised and presented in the log-compressed scale. First row demonstrates the difference between the denominators of the *W_V_* and *W_CF_* weights. Second row demonstrates the difference between the two weights.

Distributions of channel signals, particularly the signal phase, become dispersed for pixels away from the scatter, as shown in Fig. 1. In this study, we explore using the variance, another measure of the coherence of the channel data, for beamforming. Variances in channel signals for each pixel of the image are shown as a variance map in Fig. 2. An adaptive weight using this variance map is

(8) $$\begin{array}{c} W_{VN}(t)=\\ \frac{|\sum_{n=1}^{N}s_{n}(t){|}^{2}}{\sum_{n=1}^{N}|s_{n}(t)-\frac{1}{N}\sum_{n=1}^{N}s_{n}(t){|}^{2}} \end{array}$$

where the numerator is the coherent energy and the denominator is the variance. To remove element sensitivity from *s_n_*(*t*) and recover the real variance in delayed signals, the denominator additionally incorporates inverse apodisation, where *s_n_*(*t*) is divided by the element sensitivity *a_n_*(*t*). The above weight is rewritten as

(9) $$W_{V}(t)=\frac{|\sum_{n=1}^{N}s_{n}(t){|}^{2}}{\sum_{n=1}^{N}|\frac{s_{n}(t)}{a_{n}(t)}-\frac{1}{N}\sum_{n=1}^{N}\frac{s_{n}(t)}{a_{n}(t)}{|}^{2}}$$

The proposed weight is dimensionless. *W_V_* (t) can then be applied to the DAS beamformed voxel intensity *y*(*t*)

(10) $$y_{CV}(t)=W_{V}(t)y(t)$$

A key difference between this Coherence to Variance (CV) beamformer and the CF beamformer is the subtraction of the mean channel signal in the denominator. As shown in Fig. 1(b), distributions of channel signals in the centre and boundary of the main lobe (main lobe 1 and 2) are similar in amplitudes but different in phase angles. While the coherence factor reduces gradually when the pixel moves from the centre to the boundary of the PSF, variance can be close to zero at the centre of the PSF and hence creates a more well-defined main lobe peak and reduces the side lobes in the CV weighting as shown in Fig. 2.

### E Simulations

Three simulations were done to compare the image quality of the four beamformers and one simulation was done to analyse the effect of beamformers on localisation. The aforementioned 32×32 matrix array probe is simulated to image scatters, using Field II. Gaussian windowed two-cycle sinusoidal waves are set as the transmission and reception response of each element of the probe. The matrix probe was set to transmit a single plane wave. The reconstructed voxel size was 0.05×0.05×0.05 mm^3^ cube. The first simulation was imaging a scatter with a reflection coefficient of 1. The second simulation involved imaging a scatter with a reflection coefficient of 0.1. In the third simulation, five scatters were placed on the central axis of the probe and spaced equally between 15 and 25 mm from the probe surface. The five scatters were given different scattering coefficients, decreasing from 1 to 0.1 with equally spaced steps. In above three simulations, the *W_VN_* weight is additionally investigated to analyse the effectiveness of inverse apodisation; the corresponding beamformer is denoted as *C_VN_*.

Quantitative evaluations were done for the third simulation. Additive white Gaussian noise with an SNR of 0 dB and 10 dB were added to the radio frequency (RF) channel signals prior to image reconstruction respectively, to evaluate the Signal to Noise Ratio (SNR) of the four beamformers. After reconstruction, SNR is calculated by taking regions of noise only and comparing them to the averaged intensity of the centre PSF of the five targets. To quantitively compare the PSFs obtained by the beamformers, Peak Side-to-Main Lobe Ratio (PSMR) and Full Width Half Maximum (FWHM) were calculated. Two kinds of PSMR are investigated. The conventional PSMR is defined by the peak ratio of the side lobe to the corresponding main lobe, and we named it self-PSMR (SPSMR) for convenience. PSMR can also be calculated by the peak ratio of each side lobe to each different main lobe and is named cross-PSMR (CPSMR). The maximum of CPSMR is denoted as max-PSMR. In super-resolution imaging, a higher PSMR and a lower SNR give a higher possibility of mistaking side lobes and noise as single bubbles whereas a larger FWHM makes it more difficult to isolate close bubble images. The time taken to process a single frame, consisting of 1.32×10^7^ voxels, acquired with each beamformer is also listed for comparison.

In the fourth simulation, scatters were randomly placed with a uniform distribution in an imaging volume of 10×8×8 mm^3^ and a uniform distribution in reflection coefficient from 0.1 to 1. 100 scatters are generated per volume and 100 different volumes are generated. 0 dB white Gaussian noise was added to the channel signals. 3D images were reconstructed by DAS, *p*-DAS, CF and CV beamformers. The total number of localisations was kept the same for all beamformed sequences by detecting a same number of lobe peaks from each frame. Between 20 and 150 peaks with the highest intensities were taken from each frame. A localisation is counted as true positive localisation if it is within half of a wavelength of a ground truth scatter location. True Positive Rate (TPR), the ratio of true positive number to scatter numbers, and False Positive Rate (FPR), the ratio of false positive number to localisation number, are calculated to quantify the localisation performance with different beamformers.

### F Experiment

#### 1) In vitro phantom

An in-house prepared perfluorobutane microbubble solution [37] was diluted to a concentration of 6×10^6^ bubbles/ml and pumped into two Hemophan cellulose tubes (Membrana, 3M, Wuppertal, Germany) with 200±15 μm inner diameter and 8±1 μm thickness wall using a syringe pump (Harvard Apparatus, Holliston, MA, USA). The two tubes were placed almost in parallel on the probe elevationlateral projection plane and crossed with an angle of 3 degrees on the depth-lateral projection plane.

#### 2) In vivo rabbit kidney

A specific-pathogen-free New Zealand White rabbit (male, HSDIF strain, age 13 weeks, weight 2.4 kg, Envigo, UK) was sedated with acepromazine (0.5 mg/kg, i.m.) and anaesthetized with medetomidine (Domitor, 0.25 mL/kg, i.m.) plus ketamine (Narketan, 0.15 mL/kg, i.m.). Anaesthesia was maintained for approximately 4 hours by administration of 1/3 of the initial medetomidine and ketamine dose every 30 minutes. Bubbles were injected with a first bolus of 0.1 ml and following boluses of 0.05 ml.

To access the renal vasculature, the rabbits were shaved and positioned supine. Following tracheotomy, mechanical ventilation was given at 40 breaths/minute. Body temperature was maintained with a heated mat. Oxygenation and heart rate were continuously monitored. The experiment complied with the Animals (Scientific Procedures) Act 1986 and was approved by the Animal Welfare and Ethical Review Body of Imperial College London (No.P15180DF2; Date: March 17th, 2017).

#### 3) Imaging sequence

Data of the phantom and rabbit kidney were acquired with the Vantage 256 ultrasound research system (Verasonics Inc., Kirkland, WA, USA) and with the matrix array probe. As there are 1024 elements in the probe and only 256 channels in the research system, a multiplexing technique is used, in which 1024 elements are excited at the same time to transmit plane waves and four sub-apertures of a 32×32 matrix, each of which contains 32 (lateral)×8 (elevation) elements, receive signals in sequence to generate one frame. Images were acquired at a frame rate of 500 Hz without angle compounding. 2 s (1000 frames) of *in vitro* data and 3 s (1500 frames) of *in vivo* data were acquired respectively.

#### 4) SR Processing Pipeline

**Fig. 3:**
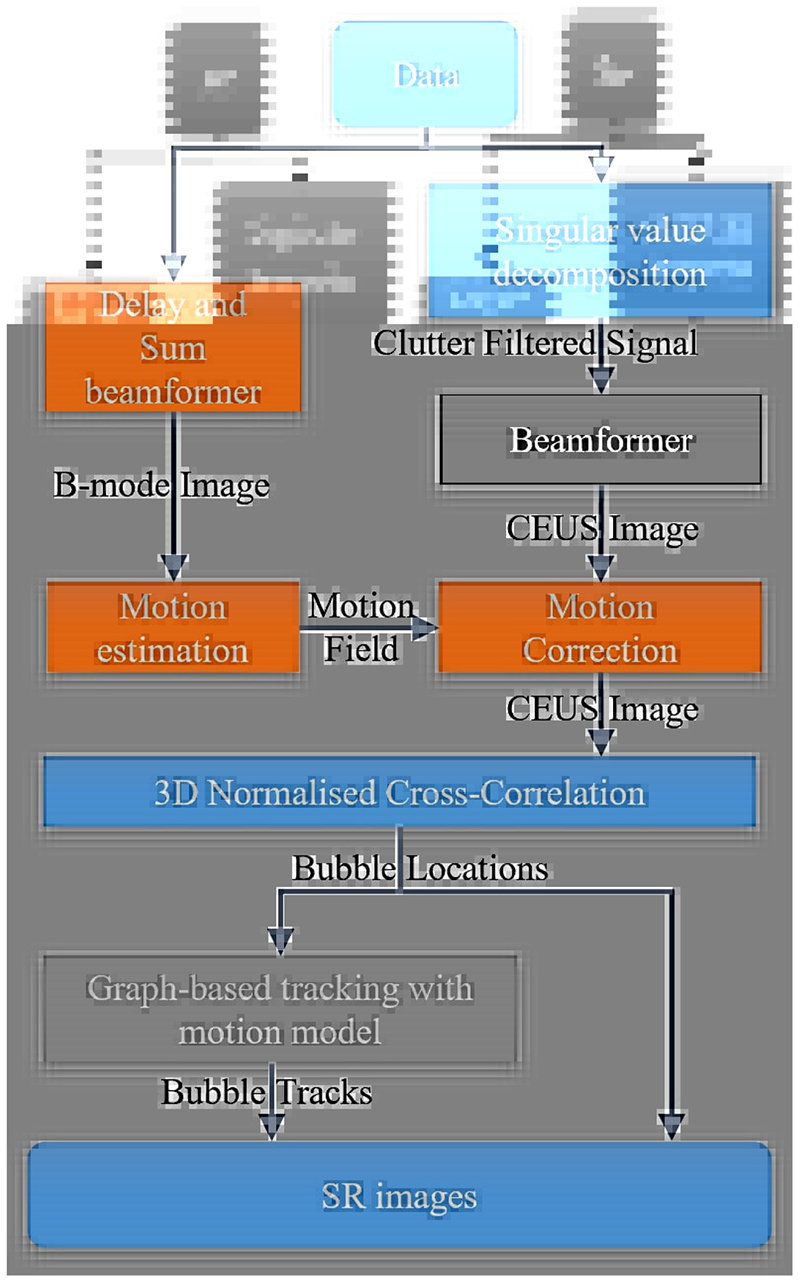
Data processing diagram. *In vitro* data go through the blue blocks and *in vivo* data additionally go through the orange blocks to correct tissue motions among frames. Only the used beamformer (black box) is different among the comparison. Comparison was done without tracking to avoid the filtering effect of the tracking. An SR blood flow video of the rabbit kidney generated with the state-of-art bubble tracking algorithm [36] is supplemented.

Data processing was conducted according to the pipeline shown in Fig. 3. Tube and tissue background signals were removed by applying singular value decomposition (SVD) to channel signals and reconstructed channel signals were then beamformed by the four techniques to obtain the contrast-enhanced ultrasound (CEUS) sequence. The *in vitro* voxel size was 0.05×0.05×0.05 mm^3^ and the *in vivo* voxel size was 0.1×0.1×0.1 mm^3^ to fit the data to the available memory. Image intensities are normalised by the maximum of each beamformed sequence. Considering beamformers can alter the dynamic range of images, [38]—[40], thresholds for background noise and side lobe removal are adjusted for DAS, *p*-DAS, CF, and CV images to achieve similar numbers of localised events for SR images. Then, comparison was done on the SR density map, based on the assumption that with the same amount of localisation events, a better beamformer is expected to provide more correct localisations and fewer wrong/false localisations.

The PSFs for each beamformer were estimated by simulations. 3D normalised cross-correlation (3DNCC) was then implemented by convolving the background-removed images with flipped PSFs [41]. Noise was further removed by thresholding the 3DNCC coefficient maps at 0.3. Peaks on the 3DNCC coefficient maps were detected by the MATLAB ‘im-regionalmax’ function and the coefficient maps were cropped by a 5×5×5 window around the peaks into small patches. Super-resolution localisation was performed by detection of the maximum in each small patch after interpolating each patch using a cubic spline and voxels 1/10 of the original size. All localised bubbles were accumulated to generate the SR bubble density map. For *in vivo* data processing, a two-stage image registration algorithm [42] was used to correct motions, which was induced mainly by animal breathing. Motion is estimated on B-mode images reconstructed by the DAS beamformer and the resulting motion fields were applied to the CEUS images reconstructed by the four beamformers.

## III Results

### A Simulation

**Fig. 4:**
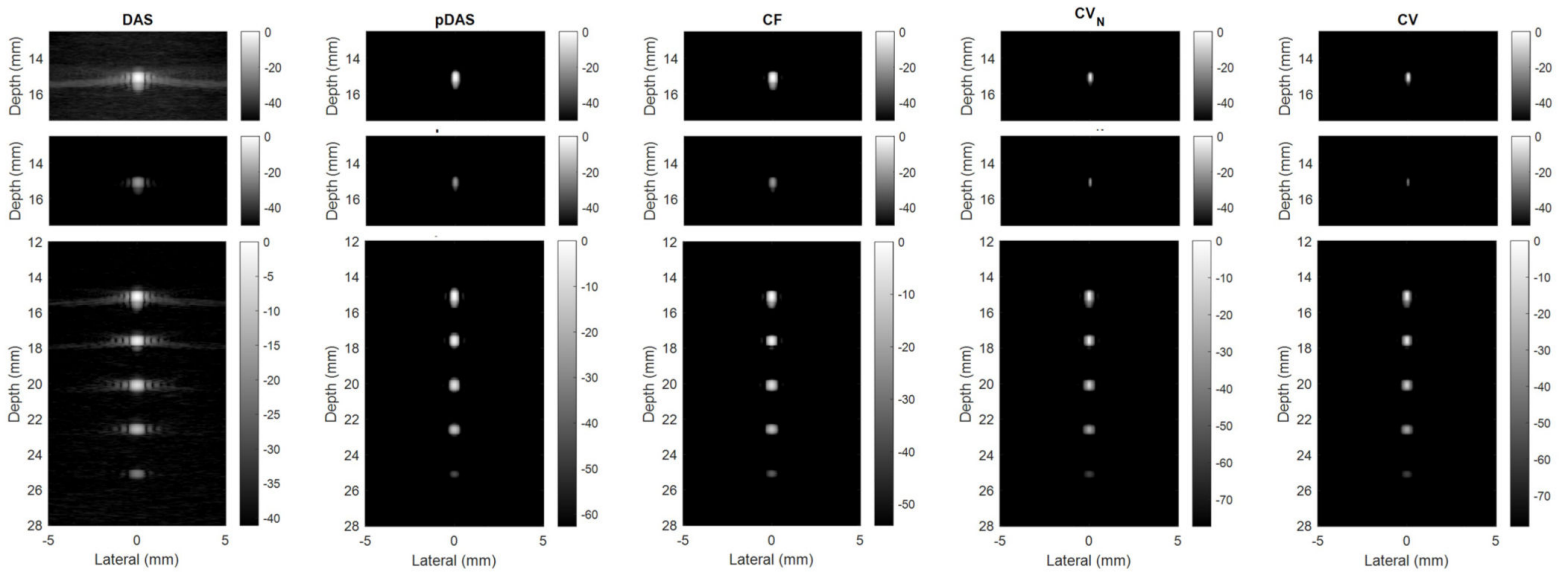
Maximum intensity projection (MIP) of 3D images reconstructed by different beamformers with channel SNR of 0 dB. The top two rows show the results of simulations imaging single scatters. The image intensities of these two rows are normalised by the intensity of the strong scatter and displayed with same dynamic range. The bottom rows show the result of simulation imaging five scatters with dynamic range set as 10% of the weakest scatter.

**Fig. 5:**
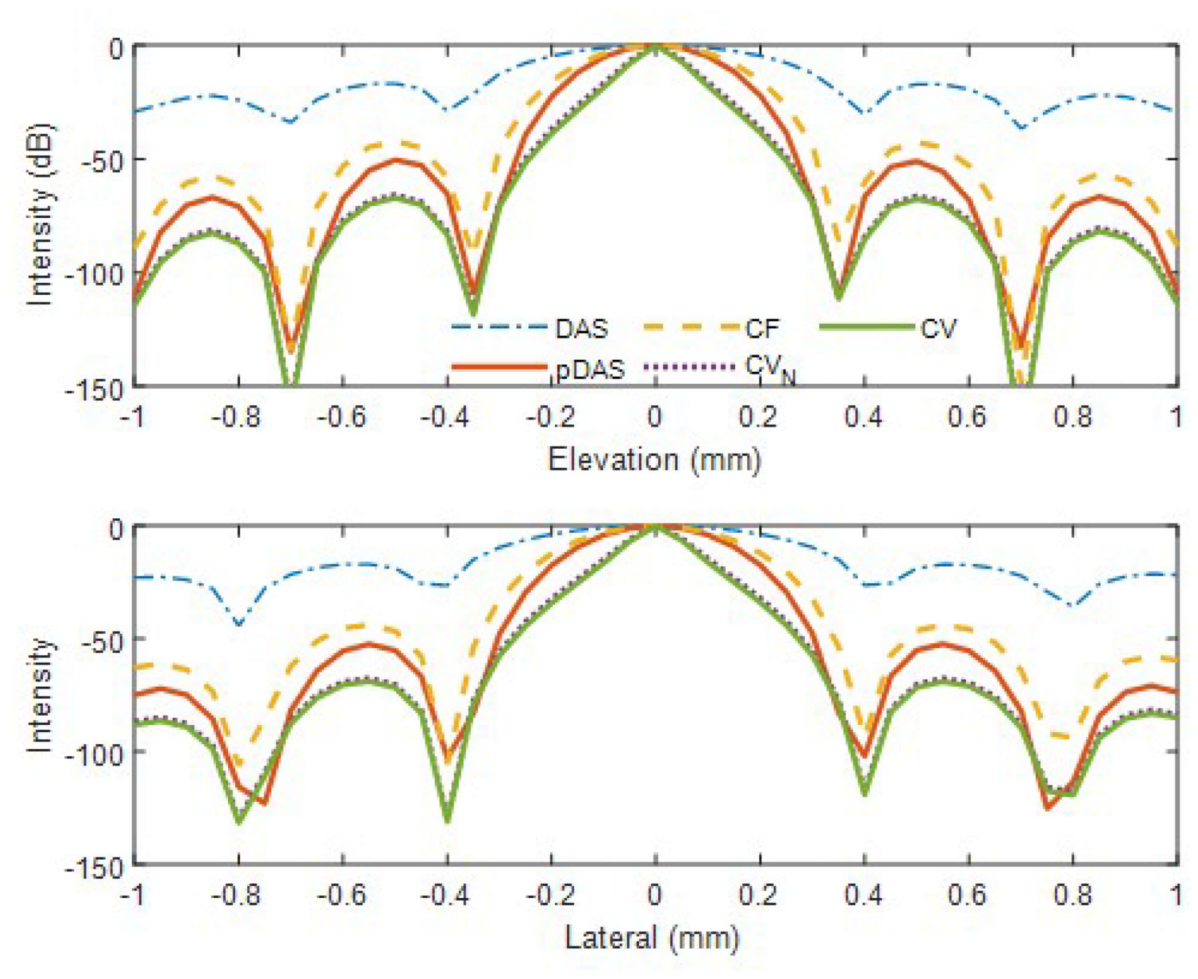
Lateral and elevation profiles of PSF reconstructed by different beamformers for one scatter.

Maximum intensity projections (MIPs) of the 3D images reconstructed with channels with SNR of 0 dB by the four beamformers are shown in Fig. 4. The top two rows show that the image intensity is linear to the reflection coefficient when strong- and weak-reflection scatters are in two frames respectively. When strong- and weak-reflection scatters are in the same frame, the intensity of the weak-reflection scatters can be also be compressed by the adaptive weight-based beamformers, as shown in the third row. While there are obvious side lobes in the DAS image, all the side lobes are almost invisible in the images beamformed by the adaptive weight-based beamformers. The image obtained by the CV beamformer is visually the best with the narrowest main lobes. The lateral and elevational profiles are shown in Fig. 5.

Quantitative metrics for the four beamformers are given in Table I and II, where the mean and standard deviations of SPSMRs and FWHMs are listed for the five scatters. Note that the max-PSMR for DAS is larger than 0 dB and is much higher than the SPSMR which means side lobes of strong-reflection scatters can be mistaken as bubbles when strong- and weak-scatters appear in the same frame. While the max-PSMR is around 3.5 dB for conventional DAS, adaptive weight-based beamformers can achieve negative values in max-PSMR at both noise levels, which means the adaptive weight-based beamformers compress the side lobes of the brighter scatter more than main lobe of the weaker scatters. Additionally, compared to lateral and elevational FWHMs obtained by DAS, CF’s have an approximately 40% reduction in the FWHMs; *p*-DAS’s achieve around a 50% reduction and CV’s reach an 80% reduction with channel SNR of 10 dB. SNRs in images are improved by the coherence-based beamformers due to the large number of channels and SNRs of the adaptive weight-based beamformers can obtain at least a 45 dB improvement over conventional DAS. In terms of computational cost, *p*DAS, CF and the variance-based beamformers require 6.9, 1.2 and 2.2 times of the DAS beamformer respectively.

**Fig. 6:**
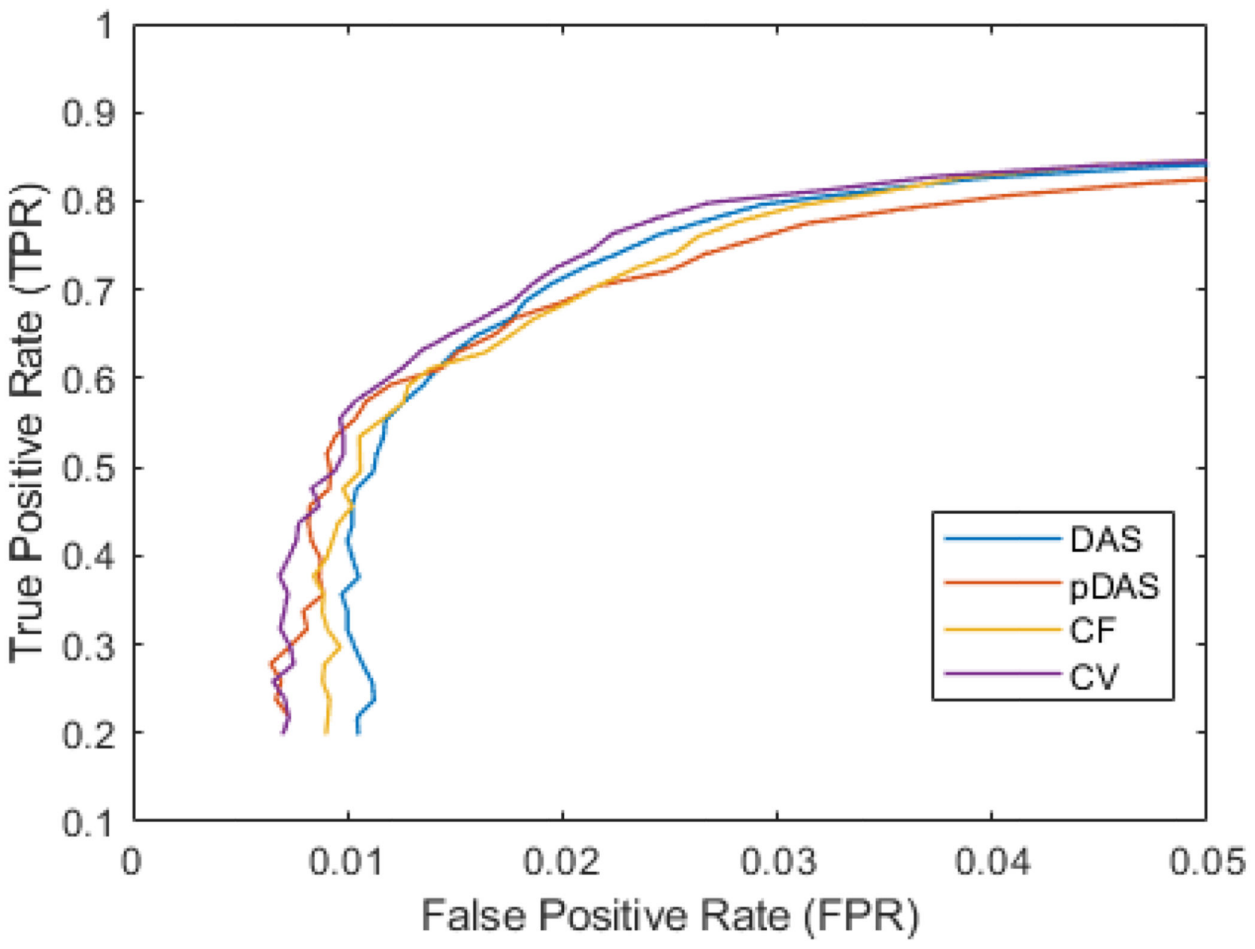
Curve plotted by TPR and FPR, with FPR lower than 0.05 presented.

A curve plotted by the TPR and FPR in the fourth simulation is shown in Fig. 6. When the curve closer to the left-top corner presents a better performance, CV gives the best performance; pDAS and CF are constantly better than DAS when the FPR is lower than 0.015. The area under the curve is 0.0605, 0.0623, 0.0616 and 0.0649 for DAS, *p*-DAS, CF, and CV respectively.

### B In vitro experiment

**Fig. 7:**
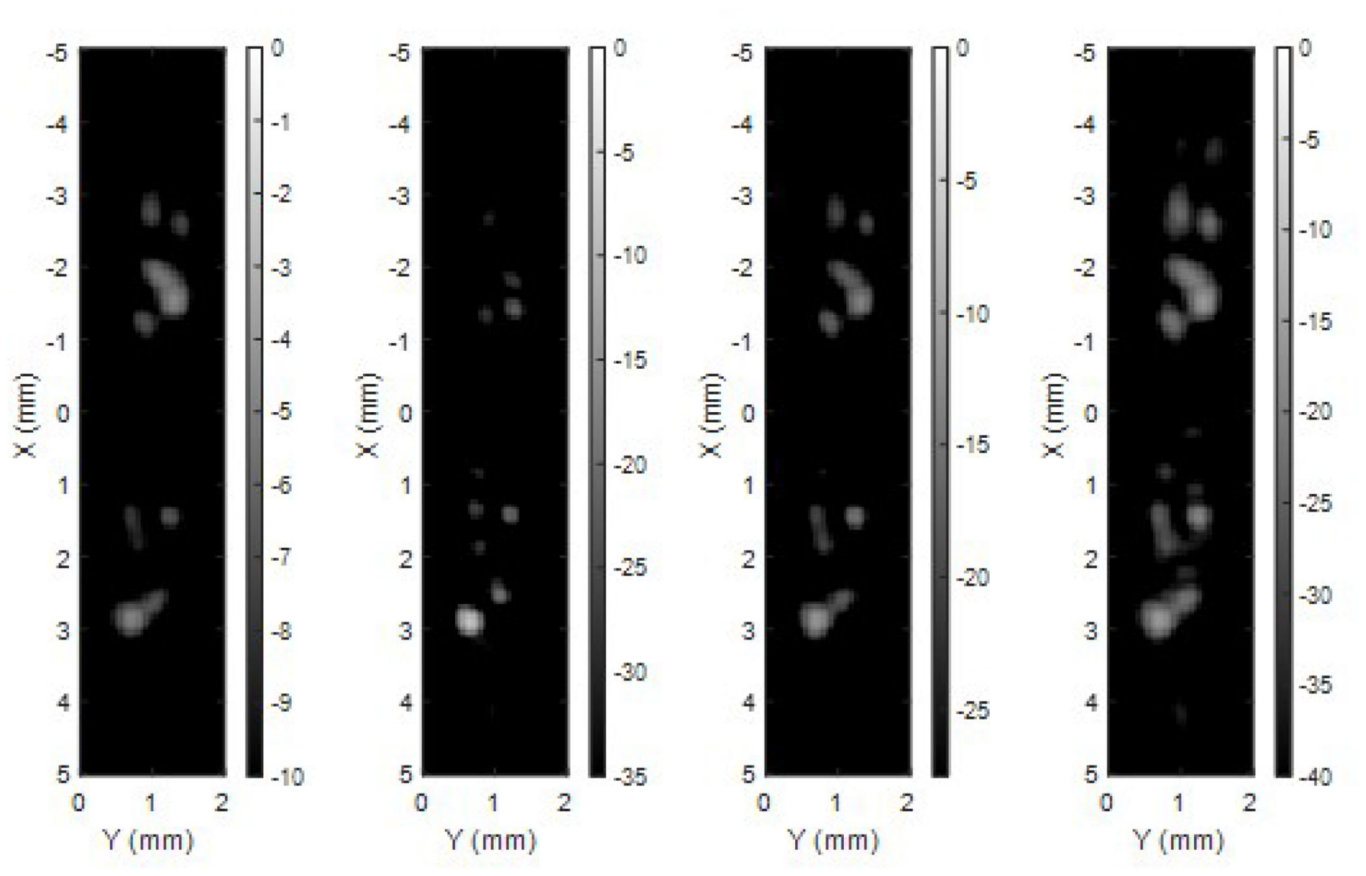
Lateral-elevation MIPs of *in vitro* bubble images beamformed by DAS, *p*-DAS, CF, and CV. The dynamic ranges used for displaying images are set by the background thresholds with which similar number of bubbles are localised from the sequences respectively.

**Fig. 8:**
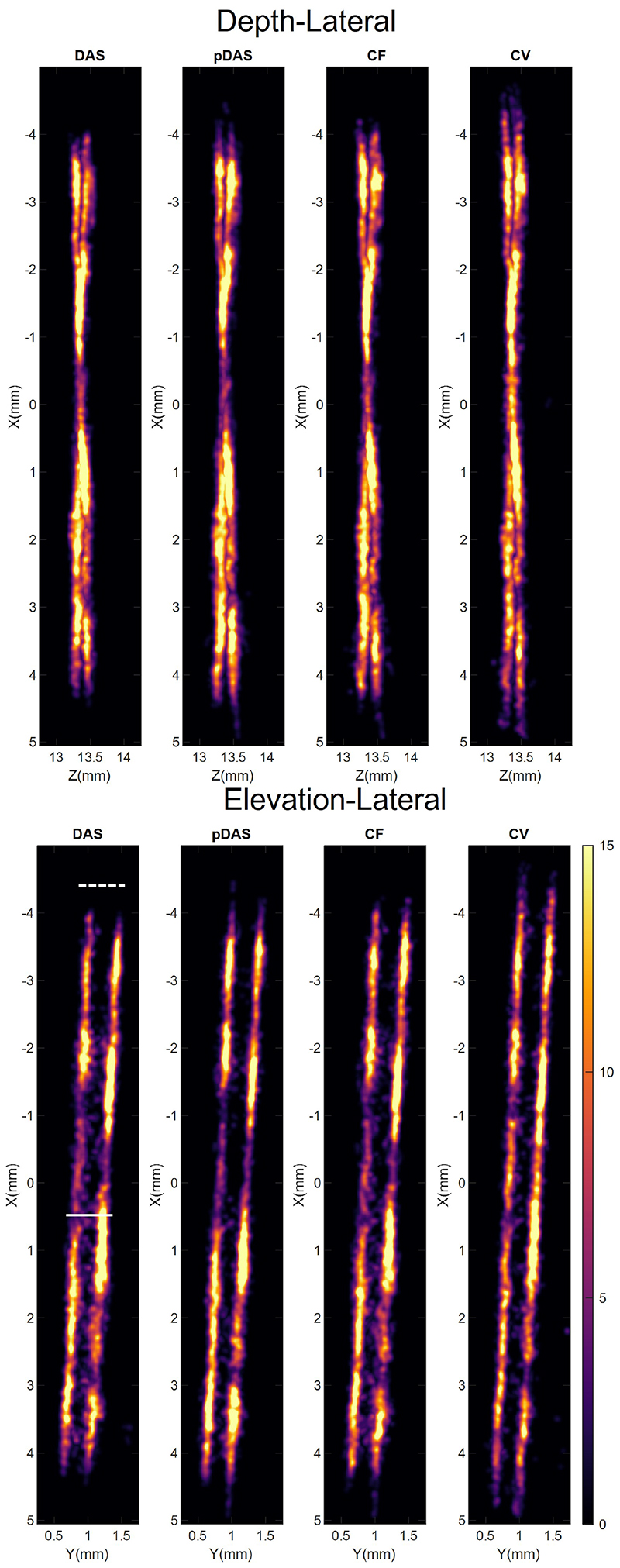
MIPs of SRUS density images obtained accumulating about 6500 bubbles detected on images reconstructed by the four beamformers.

It takes 0.047, 0.396, 0.057, and 0.096 seconds to reconstruct 9.93×10^5^ voxels per frame in the *in vitro* data by the DAS, *p*-DAS, CF, and CV beamformers respectively. 6439, 6413, 6829 and 6469 bubbles can be localised by using thresholds of -10 dB, -35 dB, -27.5 dB, and -40 dB for the DAS, *p*-DAS, CF, and CV images respectively. Bubble images reconstructed from clutter-filtered signals by the four beamformers are shown in Fig. 7 with the background removal threshold as the dynamic range for display. SRUS images obtained by accumulating these bubbles are shown in Fig. 8. As no microbubbles should be outside the tubes, the fewer localisations between the two apparent tube structures for the adaptive weight-based beamformers suggest an improved performance over the DAS beamformer, although this is not conclusive as the ground truth tube location is not known. The SRUS image obtained with the CV beamformer gives the highest signal at around 0 mm in the lateral direction where the two tubes are the closest.

**Fig. 9:**
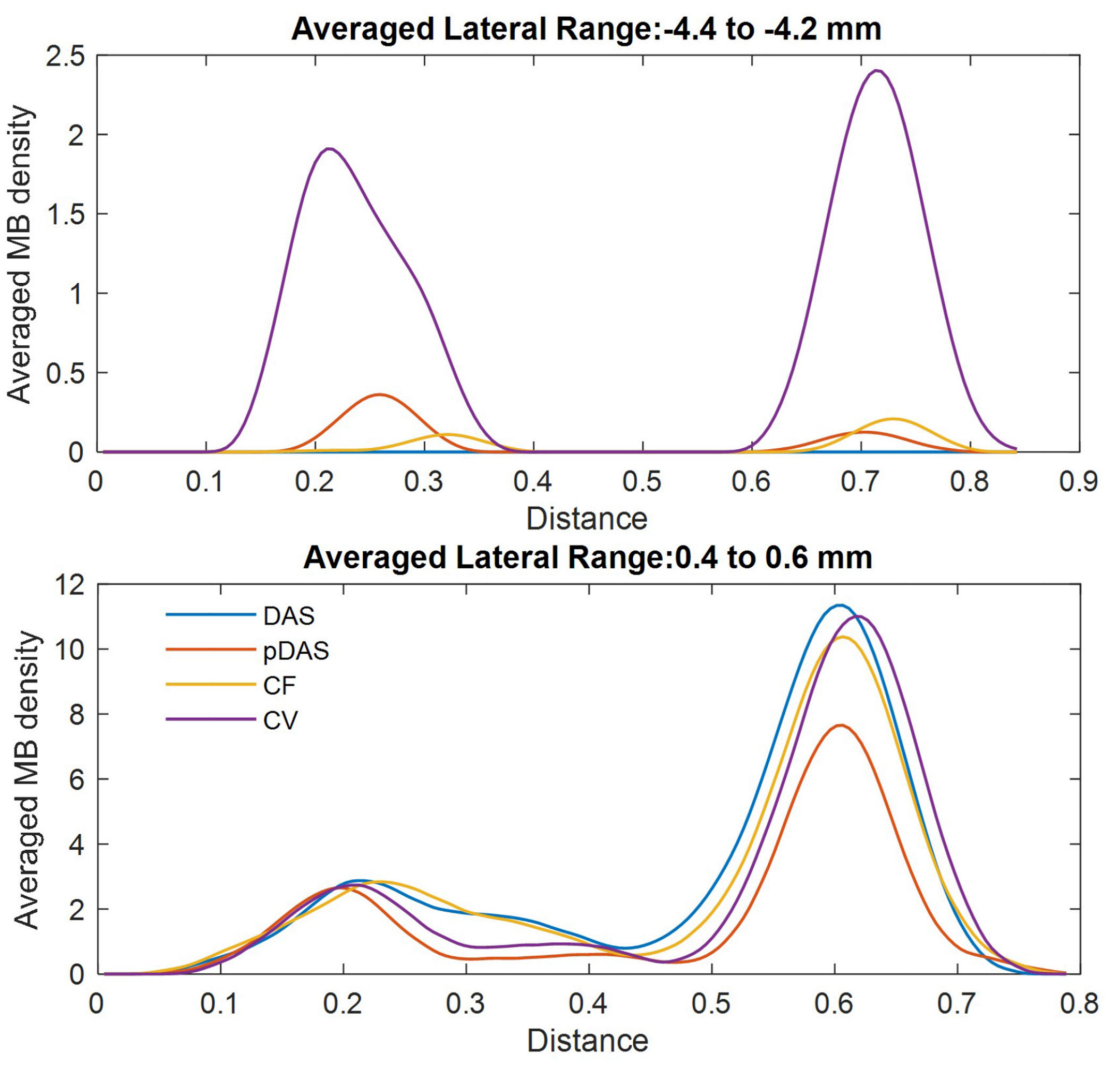
Microbubble (MB) density profiles on line across two tubes within two chosen 2D slices in Fig.8. Top: Profiles within the slice labelled by the dash line. Bottom: Profiles within the slice labelled by the solid line.

Microbubble density line profiles of the two tube cross-sections within two chosen 2D slices, each of which was labelled by a white line in the DAS image and averaged across a thickness of 1 wavelength to be smoothed, are shown in Fig. 9. For the slice from 0.4 mm to 0.6 mm in lateral, the two tubes cannot be clearly separated in profiles of the SRUS image using the DAS beamformer. In contrast, the two peaks are distinguishable in the SRUS image profiles obtained using the adaptive weight-based beamformers. For the slice from -4.4 to -4.2 mm in the *x* direction, the DAS SRUS image completely loses the tube signal, and the profile of the CV SRUS presents the highest two peaks.

### C In vivo experiment

**Fig. 10:**
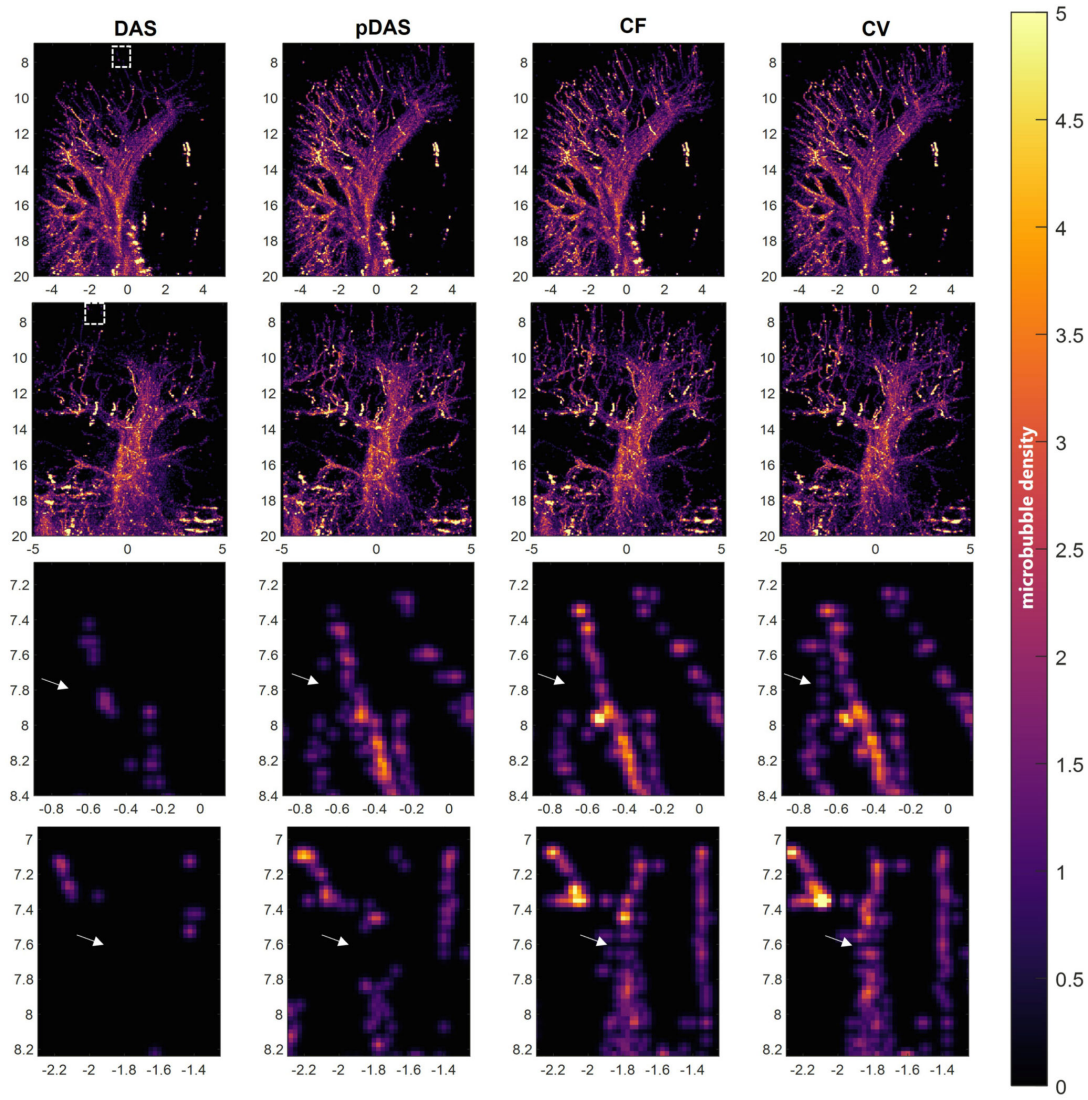
MIPs of SRUS images of rabbit kidney. The four columns correspond to the DAS, *p*-DAS, CF, and CV beamformers, from left to right. First row: lateral-depth-plane MIPs; Second row: elevation-depth-plane MIPs; Third and fourth rows: Zoomed-in images of the area in the boxes shown in the first and second rows respectively.

It takes 0.067, 0.893, 0.080, and 0.140 seconds per frame to reconstruct 1.36×10^6^ voxels in the *in vivo* data by the DAS, *p*-DAS, CF, and CV beamformers respectively. 74842, 67495, 69292 and 63660 bubbles are localised by using thresholds of -14 dB, -42.5 dB, -32.5 dB, and -35 dB for DAS, *p*-DAS, CF, and CV images respectively. MIPs of 3D *in vivo* SRUS images are shown in Fig. 10. More dense vessels in the top region and less noise in the bottom region can be detected with the adaptive weight-based beamformers, compared to those with the conventional DAS. In the magnified images in the third and fourth rows, all the adaptive weight-based beamformers provides more SRUS vascular signals than the DAS beamformer. Also, the CV beamformer generates localisations that resemble the appearance of micro vessels which are absent by the other methods.

**Fig. 11:**
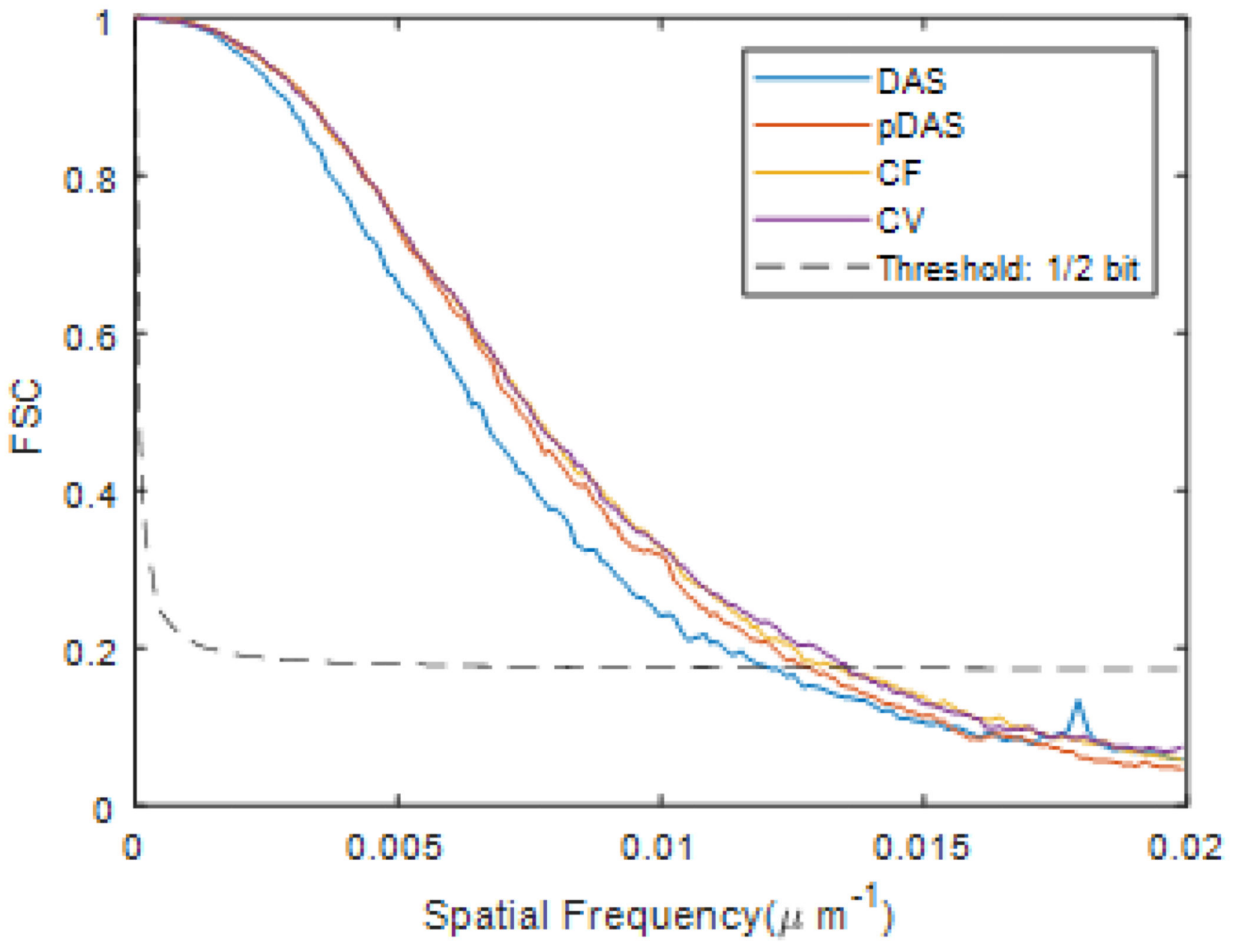
FSC curve estimated on SRUS images obtained from four beamformed sequences.

Results of Fourier shell correlation (FSC) analysis [43] between SRUS images obtained by localisation events appearing in odd and even frames are shown in Fig. 11. According to the ½ bit threshold, resolutions are measured as 83.3, 78.1, 75.2 and 74.1 μm for DAS, *p*-DAS, CF, and CV respectively, demonstrating the resolution improvement of adaptive weightbased beamformers.

## IV Discussion

In this study, advanced beamformers for 3D SRUS imaging were investigated. The results show that the adaptive weightbased beamformers can suppress the side lobes and narrow the main lobe, and are able to improve 3D SRUS images *in vitro* and *in vivo* with modest computational cost. They also show that the proposed variance-based beamformer performs best both in simulations and experiments. Compared to CV_*N*_ [44], CV generates side lobe intensity and main lobe width reduction in simulation by considering channel element sensitivity.

Improved isolation and localisation of individual bubbles in a single frame can reduce acquisition time and hence the impact of motion on SRUS imaging. While most existing SRUS studies tried to isolate bubbles in high concentration by algorithms, such as deconvolution [13], [36], [45]–[47] and normalised cross-correlation [7], [14], [15], side lobes of bright bubbles are still difficult to be distinguished from the main lobe of less bright bubbles. Using high enough thresholds or picking a few highest peaks can reduce the possibility of mistaking side lobes for SR images in practice, but discarding weak bubble signals can increase the acquisition time to achieve the same level of reconstruction. Classical DAS beamforming has limited image resolution and contrast. While the side lobes can be compressed by coherent compounding and apodisation, the improvement is at the expense of frame rate or resolution. Additionally, the steering of a matrix array transducer is limited by the appearance of grating lobes in the image, which is governed by the pitch size of the transducer, and also by the size of overlapped volume among angles, which is governed by the aperture size of the transducer.

Adaptive weight-based beamformers were investigated for improving SRUS imaging, because of their relatively low computation cost which is valuable for dealing with the large data size from matrix array probes. For example, *p*-DAS beamformer calculates the adaptive weight for each channel by using the single channel signal; the CF beamformer calculates the adaptive weight for each pixel across a single combination of the channels. It is worth noting that images in the *p*-DAS beamformer had to be reconstructed with a voxel of 0.01 mm depth to mitigate aliasing of the band-pass filtering and then down sampled to the same voxel size with the other beamformers. Denser voxels make the *p*-DAS beamformer take much more time than the other two adaptive weight-based beamformers. DMAS [26] and short-lag spatial coherence beamformers [27] also have the potential to improve 3D image reconstruction for SRUS but are not investigated in this study, as both methods need significantly higher computational cost, given the large number of channels in case of a matrix array.

We have also shown that the improvement of the adaptive weight-based beamformers do not depend on the change in data dynamic range. While the dynamic range of the image data can change when using the adaptive weight-based beamformers, we have shown that the side lobes are compressed more than the main lobe of the weak bubble signals by the adaptive weighted-based method, as shown in Fig. 4 and reflected by the max-PSMR metric. So the benefit is not from a dynamic range alternation reported previously [40].

Normalised cross-correlation is a powerful tool to detect bubbles using its spatial features rather than its signal amplitude. However, some kind of background removal thresholding as a processing step has been used in existing SR processing pipelines, which can improve the precision of localisation [7], [24]. Lower PSMR is preferred in the background removal thresholding. For the normalised data, the background removal threshold should be higher than the PSMR to avoid mistaking the side lobes as separate bubbles. Lower PSMR gives more chance to include weaker signals. With higher PSMRS, more main lobe-like signals can be seen in the adaptive weight-based images, as shown in Fig. 7.

It should be noted that SRUS processing in this study used the 3D normalised cross-correlation with the corresponding simulation PSF to localise 3D bubbles. Simulated PSFs do not consider some of the physics, such as the nonlinearity of bubbles and phase aberrations, which may explain difference between simulation and experiment. As a result, the cross-correlation between the estimated PSF and the experimental data is low and thus 0.3 is selected as the cross-correlation coefficient threshold to include enough signals for the SRUS reconstruction.

For the SRUS image of the cross-tube phantom, there should be no bubbles outside of the two tubes but the area between the tubes is not totally black due to false separations and localisations (Fig. 8). SRUS images derived from the adaptive weight-based beamformers have fewer wrong localisations than the baseline. The intensity profiles also demonstrate the benefit of using adaptive weight-based beamformers for 3D SRUS imaging to obtain more distinguishable structures and the superior performance of the CV beamformer, which gives the longest presented tube images. While we aimed to conduct the comparison between the different beamforming methods using the same number of localisations, in practice the exactly same number is challenging to achieve and, in this case, the chosen threshold of DAS allowed it to have more localisations from the *in vivo* data than the other methods. The adaptive weighted-based beamformers give more localisations than the DAS beamformer and the CV beamformer gives the most localisations in vessels close to the probe.

The appropriate imaging volume rate for tracking bubbles depends on the bubble concentration and blood flow speed. A higher volume rate would generally make the bubble tracking less challenging, but with increased amount of data and computation requirement. A previous study used 750 Hz volume rate with 5 compounding angles when studying a similar volume [24]. In this study a volume rate of 500 Hz is empirically high enough for the tracking of bubbles in the rabbit kidney with our developed feature-motion-model tracking algorithm [36].

## V Conclusion

This study compares four coherence-based beamformers for 2D US matrix array probes to improve 3D SRUS imaging. Simulations and experiments verify the superiority of the adaptive weight-based beamformers over the DAS beamformer for single-plane-wave transmission. The proposed CV beamformer produces the lowest side lobes and narrowest main lobe in simulations and generates the best *in vitro* and *in vivo* SRUS images with a 3-second acquisition.

## Figures and Tables

**Table I:** Quantified Metrics For Four Beamformers At 0 dB SNR In Channels.

	DAS	*p*-DAS	CF	*CV_N_*	CV
SPSMR(dB)	-15.6±1.8	-52.7±1.2	-41.7±3.1	-58.8±12.6	-59.9±13.8
Max-PSMR(dB)	3.8	-9.4	-10.1	-12.3	-15.0
Lateral FWHM(mm)	0.552±0.079	0.278± 0.049	0.325±0.052	0.188±0.111	0.179±0.116
Elevation FWHM(mm)	0.503±0.071	0.251±0.047	0.296±0.048	0.170±0.102	0.163±0.106
SNR(dB)	27.4	100.7	74.1	84.9	86.8
Processing Time(s)	0.68	4.83	0.82	1.49	1.49

**Table II:** Quantified Metrics For Four Beamformers At 10 dB SNR In Channels.

	DAS	*p*-DAS	CF	*CV_N_*	CV
SPSMR(dB)	-16.1±1.2	-52.7±1.1	-42.8±1.5	-67.9±8.5	-70.9±10.6
Max-PSMR(dB)	3.4	-26.6	-20.4	-32.4	-34.9
Lateral FWHM(mm)	0.557±0.086	0.279± 0.051	0.328±0.056	0.126±0.084	0.115±0.087
Elevation FWHM(mm)	0.506±0.075	0.250±0.046	0.296±0.049	0.114±0.075	0.105±0.077
SNR(dB)	37.0	113.3	85.3	98.3	101.0
Processing Time(s)	0.69	4.86	0.81	1.50	1.51
